# Emotional processing is not enough: relations among resilience, emotional approach coping, and posttraumatic stress symptoms among combat veterans

**DOI:** 10.3389/fpsyg.2024.1354669

**Published:** 2024-06-04

**Authors:** Shai Shorer, Michael Weinberg, Lihi Cohen, Doron Marom, Miri Cohen

**Affiliations:** ^1^School of Social Work, Faculty of Social Welfare and Health Sciences, University of Haifa, Haifa, Israel; ^2^B’Shvil, Yahud, Israel; ^3^Psychology Department, University of Haifa, Haifa, Israel

**Keywords:** combat, coping, emotional approach coping, posttraumatic stress, resilience, veterans

## Abstract

Combat soldiers are exposed to various potentially traumatic events and face high risk of developing military-related psychopathology, such as depression, posttraumatic stress and grief (PTSS). However, a strong body of research shows that resilience is the default in the aftermath of trauma and indeed, many veterans do not develop high symptomatic levels. To explicate this inconsistency, the current study examined the associations among PTSS, resilience, and patterns of emotional-approach coping. A sample of 595 male combat veterans filled out questionnaires on trauma exposure, PTSS, depressive symptoms, resilience, and emotional-approach coping. Their data were analyzed using structural equation modeling path analysis. Participants reported high exposure to potentially traumatic events during service. Mean scores were high for resilience and relatively low for PTSS and depressive symptoms; 13% had a clinical level of posttraumatic stress disorder. Structural equation modeling revealed that emotional-approach coping strategies mediated the relationship between resilience and PTSS. However, emotional expression was associated with lower PTSS levels, whereas emotional processing was associated with higher PTSS levels. These results suggest that although emotional-approach coping was related to higher resilience, emotional expression (an intrapersonal coping strategy) might have a more positive effect than self-oriented emotional coping strategies. Providing veterans with supportive opportunities and a wider repertoire of emotional coping skills might enhance their well-being, reduce postservice emotional distress while not harming veterans’ resilience levels.

## 1 Introduction

Exposure to potentially traumatic events (PTEs) and their aftermath is often an inevitable part of combat service for many soldiers and veterans around the world (Korte et al., 2020). Soldiers exposed to combat are at high risk of developing posttraumatic stress symptoms (PTSS) and presenting various levels of posttraumatic stress disorder (PTSD). Prevalence rates of PTSD in veterans have been reported as varying from relatively low (1.09%) to very high (34.84%), depending on demographic, social, life experience, and exposure-related factors (Xue et al., 2015) and the veterans’ homecoming conditions (Boscarino et al., 2018). Given this wide range, Fulton et al. (2015) performed a meta-analysis and found that 23% of American soldiers who participated in recent military campaigns experienced substantial PTSD levels. Although these estimations vary based on the choice of estimation tools and cohort (Hoge et al., 2014), they still reflect a major condition that affects a large part of the veteran population. Moreover, prior research reported that exposure to military trauma was associated with other emotional disorders, such as depression (Yancey et al., 2023), which affects an estimated 9–16% of American veterans (Karstoft et al., 2020; Inoue et al., 2023). Similar rates were reported among veterans of other countries, such as Australia (Carra et al., 2022), Canada (Thompson et al., 2019), and Israel (Shorer et al., 2023).

It is well established that the substantial presence of PTSS among military veterans has multiple causes related to precursor factors, the nature of the traumatic exposure, and its aftermath (Korte et al., 2020). Military-related PTEs are often characterized by unique and high-level exposure, such as facing ongoing danger or witnessing injuries and death of friends. Exposure to PTEs during military service is also unique due to postservice factors, such as confusion of self-identity between being a soldier and a citizen; dominance of masculine characteristics that demand repressing of emotions, most notably weakness and vulnerability (Mobbs and Bonanno, 2018); and experiences of loneliness, isolation, separation from loved ones, and living for long periods in uncertain and unpredictable conditions (Solomon, 2020). However, the inconsistency of this high risk of developing PTSS and the fact that most veterans do not develop military-related psychopathology (Porter et al., 2017) demand further study (Bonanno, 2021).

Aiming to fill this gap, the current research examined the relationships among the personal resource of resilience (Connor and Davidson, 2003), considered a pretraumatic factor; emotional-approach patterns (Stanton et al., 2000a), a peri- and posttraumatic factor; and PTSS and depression, being the consequences of the complex dynamics of these factors.

Resilience is considered to be a personal resource related to better psychological reactions to stressful life events (Bonanno, 2021). Although definitions of resilience as a psychological construct vary (Denckla et al., 2020), scholars generally agree that resilience is not a stable characteristic. Rather, it reflects the dynamics of different peritraumatic factors that comprise and shape the process of coping with the aftermath of traumatic exposure (Bonanno, 2021). It consists of a combination of psychological, physiological, and behavioral characteristics that enable individuals to resist, cope with, and recover in the face of adverse life experiences (Masten and Powell, 2003). Based on the accumulated body of research in this field, researchers have suggested that resilience can be taught and practiced. It is the product of an ongoing process of individual’s evaluation of the situation they face, choosing resilience-enhancing coping strategies among those available, implementing them, and constantly evaluating their effect to make necessary adjustments to improve their coping (Bonnano et al., 2023). This approach considers resilience as a state of mind that can and should be constantly used. It can be evaluated at a specific point in time (Connor and Davidson, 2003), and research has shown high levels of resilience among veterans associated with relatively low levels of PTSD (compared to the general population), despite experiencing multiple PTEs during military service (Galatzer-Levy et al., 2018). Studies have repeatedly examined this relationship, highlighting the notion that PTSS, resilience, and growth often coexist (Zerach et al., 2013). For example, research with a nationally representative sample of U.S. veterans found that most participants presented moderate to high levels of resilience, along with relatively low levels of PTSD (Fogle et al., 2020). This comprehensive study described various important relations among veterans’ active coping methods, distress, and well-being. The study concluded that veterans who were more involved in therapy and social interactions, accepted their emotional and behavioral challenges, and engaged in efforts to solve them presented lower distress over time. Resilience, as a personal resource, was shown to be related to use of more efficient coping strategies in the face of stressors (Bonnano et al., 2023). Moreover, difficulty with emotional clarity (e.g., understanding one’s emotions) and lack of emotional regulation strategies were found to be prominent in the development of PTSD and depression symptoms, two common emotional reactions following traumatic injury (Korte et al., 2020). Notably, lack of emotional clarity was associated with PTSD symptoms of hyperarousal and negative alterations in mood and cognition, overall PTSD symptom severity, and depression levels (Timmer-Murillo et al., 2023). PTSS and depression often co-occur and have been found to evolve in tandem (Armenta et al., 2019). The current study further explored these trajectories.

Theoretical models of coping with stressful situations define coping as the behavioral and cognitive means that individuals use to deal with stressors (Lazarus and Folkman, 1984), and it has been classified into different modes (e.g., problem-focused and emotion-focused coping). In recent years, leading stress and coping researchers have proposed the emotional-approach coping classification (Stanton and Low, 2012). The concept of emotional-approach coping was developed in response to Lazarus and Folkman’s conceptualization of emotion-focused coping (Biggs et al., 2017). A major critique of their model is that it aggregates diverse strategies and does not distinguish between ineffective strategies of disengagement and avoidance, which usually increase emotional distress, and other efficient emotion-focused strategies, such as emotional processing and expression (Stanton et al., 2000a; Carver and Connor-Smith, 2010).

Emotional-approach coping involves processing and expressing emotions in response to stressful life experiences or exposure to adversities (Stanton et al., 2000a). This process is commonly divided into two strategies: emotional processing, which consists of attempts to acknowledge, explore, and understand emotions; and emotional expression, which involves verbal or nonverbal efforts to express or share emotional experiences. Previous studies reported that emotional-approach coping, combining emotional expression and processing, was associated with lower psychopathological symptoms and better adjustment to stressful situations (Stanton and Low, 2012; Juth et al., 2015; Hoyt et al., 2020). Nevertheless, studies also showed distinctive associations of emotional expression and processing with psychological factors. For example, emotional expression was associated with lower distress indexes, whereas diverse results were reported regarding the effect of emotional processing. Scholars suggested that whereas emotional expression helps relieve emotional load and is often related to other people, emotional processing may involve personal ruminations on negative cognitions and emotions (Stanton et al., 2000a; Marroquin et al., 2016). However, the effect of emotional expression and processing may depend on time, gender, and situation (Stanton and Low, 2012; Juth et al., 2015; Hoyt et al., 2020).

Only a few studies have examined the effect of emotional-approach coping on PTSS and emotional distress among trauma-exposed individuals (Hassija et al., 2012; Fishbein et al., 2022). The current study aimed to fill this theoretical gap. In a study with cancer survivors, emotional-approach coping was associated with lower cancer-related trauma symptoms and mediated the effect of acceptance and commitment therapy on decreased symptoms (Fishbein et al., 2022). Another study, involving 209 trauma-exposed U.S. veterans, found that emotional expression was associated with lower PTSD and depression symptoms, whereas emotional processing was not (Hassija et al., 2012).

A study examining the complex relations between resilience and emotional-approach coping found that individuals who shared more of their experiences tended to express higher emotional resilience and have lower rates of PTSD symptoms and emotional distress (Yeung and Chow, 2019). Moreover, resilience was found to moderate the relations among combat exposure, intrusive ruminations, and PTSD levels at various levels of trauma exposure (Blackburn and Owens, 2016). Yet veterans may refrain from sharing their distress or seeking help for emotional issues. In other words, they tend to make partial use of their potential resilience resources (Lahad, 2017; Mobbs and Bonanno, 2018). This trend might be the consequence of shame regarding their combat experience and how they cope with its aftermath, because the military’s masculine and stoic social norms discourage the expression of emotions perceived as weakness (Gaudet et al., 2015).

Israeli citizens are obliged to complete army service of 2–3 years at age 18 (Knesset, 1986), and male Israeli veterans usually continue to serve in the reserve forces until age 40 (Knesset, 2008). Due to this state’s ongoing struggle for security and its small size, Israeli veterans are at risk of multiple PTEs and retraumatization (Solomon, 2020). A recent report indicated about 5,700 of Israel’s emotionally injured veterans were recognized by the Ministry of Defense’s Rehabilitation Wing as dealing with PTSD (Yechimovitch-Cohen, 2022). However, this rate is expected to grow due to the state’s recent violent conflicts (Ministry of Health, 2023). Because about 100,000 soldiers are recruited and discharged each year, this figure probably underestimates the number of Israeli veterans experiencing substantial levels of PTSS.

The current study examined the relationships among resilience, emotional-approach coping strategies, and PTSS and depressive symptoms among Israeli veterans. Specifically, we examined the associations between resilience and levels of PTSS and depression, along with the mediating role of emotional-approach coping strategies (emotional expression and processing) in the association of these factors. The cohort of this study was a unique nonclinical group of people with prior PTEs who were still at risk of exposure to additional PTEs (because most of them served as reservists); they were in touch with both their natural support systems and a military-related, assumedly supportive environment (peer combatants).

## 2 Materials and methods

The study was approved by the affiliated faculty’s board of ethics (No. 266/21).

### 2.1 Participants and procedure

The current paper presents the results of the first wave of a longitudinal study on the impact of military service on Israeli combat veterans who participated in a nature-assisted group intervention for processing military-related PTEs called B’Shvil. B’Shvil is a nonprofit organization that aims to help Israeli veterans who served in military units of all forces to process their combat experience, address difficulties in the bidirectional transition between army and civilian life, and facilitate postservice psychological growth (Shorer et al., 2023). Teams of veterans (e.g., squads, companies, platoons) voluntarily participate in this intervention, usually around 5–10 years after their release from mandatory service (and the continuation of reserve service). The group intervention is usually operated as a 10-day retreat in nature settings (to learn more about this intervention, please refer to https://www.bshvil.org/english). Overall, including personal and group meetings before and after the retreat, participants are followed by B’Shvil facilitators for 6 months. A few main therapeutic components are highlighted in this recreational intervention: mind–body relations in the face of trauma; personal and group psychological resilience; and nature’s healing power.

The full research project will examine participants’ condition at three points: before the intervention, after the invention, and at follow-up. Participants completed informed consent forms and self-reported questionnaires at the beginning of the group intervention.

The study sample consisted of 595 male veterans who served in various combat units and military forces (e.g., infantry, armory, navy, air force). Overall, 1,033 veterans participated in the intervention between February 2021 and October 2022 (when data were collected). In this sample, 640 (62%) participants filled out the study questionnaire; 17 participants were excluded because they did not complete the questionnaire. Although women comprise about 40% of Israeli military personnel and 19% of them serve as combatants (Shafran Gittleman, 2020), only a dozen women participated in these groups and only six of them completed the questionnaire. Hence, their data were excluded from the current study. An *a priori* sample size calculation for SEM: Statistical Equation Modeling (Soper, 2024) indicated that a sample of 400 participants is needed to detect an effect size of 0.10 with desired power of 0.80, seven observed variables (including controlling for confounding variables), and one latent variable.

Table 1 shows the demographic characteristics of the participants. Their mean age was 32.9 (*SD* = 6.4); 36% were single and the rest were either married or in a relationship; and 97% were engaged in either a full-time job or academic studies. Most participants (93%) reported exposure to PTEs, and 11% were injured during their army service.

**Table 1:** Participants’ demographic data.

	*n* or *M*	% or SD	Range
Age	31.72	5.20	24–47
**Marital status**^a^			
In relationship	198	33.7	
Single or divorced	389	66.3	
Education	14.58	2.06	10–22
**Employment**			
Work or study	579	97.30	
Not working or studying	16	2.70	
**Income**^a^			
Below average	170	28.50	
Average	111	18.70	
Above average	193	52.80	
**Religiosity**			
Religious	193	32.40	
Secular	402	67.60	
Injury during service	44	11.80	
Exposure to PTE	551	92.60	

PTE, potentially traumatic events.

a Percentage was calculated from the actual number of responses.

### 2.2 Measures

Demographic data and army service details were collected, including what roles participants held during their army service (active combat, supporting active combat, etc.).

Trauma exposure was examined using Life Events Checklist for DSM-5 (Weathers et al., 2013), which was shortened to fit this study population. Six items were eliminated to avoid redundancy. These items related to occupational and recreational injuries, risk of toxification, nonspecific sexual abuse, captivity, exposure to general human suffering, and exposure to sudden death. The final list of events included exposure to combat, other military-related threats to their life or military-related physical injury, civil violence that included trauma, terror attack, car accident, life-threatening natural disaster, life-threatening illness, and other types of traumatic events.

PTSS was evaluated using the Hebrew version of the PTSD Checklist for DSM-5 (PCL-5; Weathers et al., 2013; translated by the Israeli Ministry of Defense). This 20-item questionnaire features a 5-point Likert scale ranging from 0 (not at all) to 4 (extremely) regarding experiences of PTSD symptoms during the last month. For example: “In the past month, how much were you bothered by: Repeated, disturbing, and unwanted memories of the stressful experience?” Participants’ scores were calculated as a combination of their total score (range: 0–80) and whether they currently experienced symptoms in four PTSD clusters. This questionnaire was found to have high internal reliability (Blevins et al., 2015). Initial research suggested a cutoff score of 31–33 as indicative of probable PTSD across samples (National Center for PTSD, n.d.). Correlations between the subscales ranged from 0.71 to 0.93. In the current study, Cronbach’s alpha was 0.96.

Depressive symptoms were evaluated using the 6-item depression subscale of the Brief Symptom Inventory-18 (Derogatis, 2001). Participants were asked to rate certain feelings during the previous 7 days on a 5-point scale from 0 (*not at all*) to 4 (*extremely*). The inventory has been translated into Hebrew and is widely used. A mean score was calculated. In the current study, internal reliability (Cronbach’s alpha) for the 18-item questionnaire was 0.95; for the six depression items, it was also 0.95.

Resilience is a multidimensional, context-related, complex phenomenon. Hence, its assessment is complicated and context related (Satapathy et al., 2022). In the current research, resilience was evaluated using the Connor-Davidson Resilience Scale (Campbell-Sills and Stein, 2007), a widely validated and used self-report questionnaire that evaluates perceptions of self-efficacy and adaptability in the face of stressful situations. This tool features 10 personal resilience statements evaluated on a 5-point Likert scale from 0 (*not at all*) to 4 (*strongly agree*) regarding the prior month. For example: “I am able to handle anything that happens in my life.” A sum score was calculated (range = 0–40). This tool was chosen because it examines the relevant resilience characteristics of adult participants, was previously used to evaluate veteran populations (e.g., Green et al., 2014; Hughes et al., 2018), and was found to have good internal reliability of 0.86 (Smith et al., 2019). In the current study, Cronbach’s alpha was 0.89.

Emotional-approach coping, consisting of emotional processing and emotional expression subscales (Stanton et al., 2000a,b), was used to evaluate participants’ emotional coping mechanisms. We used a short version of this tool—five items from the emotional expression subscale (e.g., “I allow myself to express my feelings”) and eight items from the emotional processing subscale (e.g., “I take time to understand what I really feel”). Participants rated their answers on a 5-point Likert scale (1 = *not at all*, 5 = *very much*). A sum score was calculated for each subscale (range: 8–40). We used a Hebrew translation of this questionnaire (Cohen and Numa, 2011). This tool was found to have good internal reliability in previous studies (e.g., Cohen and Numa, 2011; Marroquin et al., 2016). Internal reliability for emotional processing and emotional expression was 0.72 and 0.82, respectively (Stanton et al., 2000b). In the current study, Cronbach’s alpha was 0.94 for emotional processing and 0.93 for emotional expression.

### 2.3 Statistical analysis

Data were collected using the Qualtrics platform and later organized for statistical analysis using SPSS 27.0. Descriptive statistics and preliminary analyses were performed in SPSS. The research model was processed using a structural equation modeling path analysis with IBM AMOS 27.0, with the goal of assessing direct and indirect pathways from resilience to PTSS. The placement of the variables in the path analysis was based on the stress and coping model (Lazarus and Folkman, 1984), suggesting that the effect of resilience as a personal resource on psychological outcomes of stressful encounters is mediated via coping strategies. Six cases of missing education data were imputed using variable means. Model fit was assessed using the following indexes: chi-square and normed chi-square tests to assess the model’s overall fit and parsimony; comparative fit index (CFI), Tucker-Lewis Index (TLI), and normed fit index (NFI), which are incremental fit indexes; and root mean-square error of approximation (RMSEA) and its confidence interval (CI), which measures the discrepancy per degree of freedom and indicates the model’s absolute fit. To increase understanding of the mediation paths, the unstandardized indirect and total effects of the specific paths were evaluated using bootstrapping (5,000 bootstrap samples) via the user-defined estimands option.

## 3 Results

Table 2 shows means, standard deviations, and correlations between the study variables. Mean levels of PTSS were low but ranged widely, from 0 to 70. In addition, 66% of the participants reported very low PTSD levels (PCL ≤ 15), 21% described low to subthreshold PTSD levels (15 ≤ PCL ≤ 33), and a small yet substantial minority (13%) described clinical PTSD levels (PCL > 33). Levels of depressive symptoms were also low. Moreover, participants reported high levels of resilience, although scores varied from 0 to 40. Mean levels of emotional processing were high and substantially higher than levels of emotional expression. PTSS and depression were negatively associated with resilience. Emotional expression and emotional processing were positively associated with resilience. However, only emotional expression, which involves verbal or nonverbal efforts to express and share emotional experiences, was significantly and negatively associated with PTSS and depression. On the other hand, emotional processing, which consists of attempts to acknowledge, explore, and understand emotions, was not significantly associated with PTSS or depression.

**Table 2:** Means, standard deviations, and Pearson correlations of the study variables.

	*M* (SD)	1	2	3	4
1. PTSS	14.19 (15.60)				
2. Depressive symptoms	3.57 (4.54)	0.84*			
3. Resilience	32.97 (6.66)	−0.44**	−0.43**	–	
4. Emotional processing	27.23 (7.53)	0.01	0.08	0.12*	
5. Emotional expression	15.10 (4.86)	−0.13*	−0.13*	0.29**	0.58*

PTSS, posttraumatic stress symptoms.

*p < 0.05, **p < 0.001.

**Figure 1:**
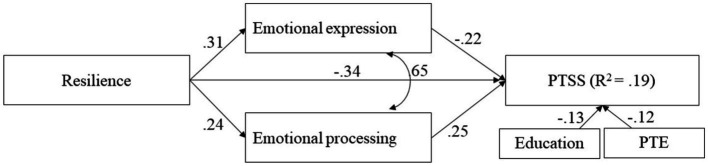
Path analysis for direct and mediated associations between resilience and PTSS. Path analysis for resilience, emotional expression, and processing and PTSS. Model also includes education and PTE as controls. Standardized estimates are shown. PTSS, posttraumatic stress symptoms; PTE, potentially traumatic events (*p* < 0.001).

Due to the high association between PTSS and depression and their similar associations with the other study variables, the consequent analysis was conducted only with PTSS. Prior to assessment of the direct and indirect associations among resilience, emotional-approach coping, and PTSS via path analysis, we examined what background variables should be controlled. Physical injury that occurred during army service, exposure to PTEs, and years of education were significantly associated with levels of PTSS. However, all injured participants also reported exposure to PTEs; therefore, exposure and education variables were added to the study model. The study model was examined with path analysis (Figure 1), in which emotional processing and expression were defined as mediators of resilience and PTSS, whereas injury and education were control variables. The fit indexes of the model were good: χ^2^(7) = 5.11, *p* = 0.65; NFI = 0.99, CFI = 1.00, TLI = 1.01, RMSEA = 0.00, 95% CI [0.00, 0.04]. The model showed that after controlling for exposure to PTEs and years of education (which were associated with PTSS), resilience was directly and negatively associated with PTSS (direct effect: *b* = −0.79, SE = 0.09, 95% CI [−1.06, −0.54]; total effect: *b* = −0.35, SE = 0.04, 95% CI [−0.42, −0.26]). Emotional expression and emotional processing were also directly associated with PTSS, showing a negative effect for emotional expression (*b* = −0.21, SE = 0.16, 95% CI [−0.31, −0.12]) and a positive effect for emotional processing (*b* = 0.25, SE = 0.10, 95% CI [1.00, 0.37]). Further analysis using bootstrapping indicated that the indirect and total effects for both paths between resilience and PTSS were significant (Table 3).

**Table 3:** Summary of total, direct, and indirect effects of resilience on PTSS.

	Effect					
	Total		Direct		Indirect	
	*b* (SE)	95% CI	*b* (SE)	95% CI	*b* (SE)	95% CI
Resilience	−0.81* (0.04)	−0.42, −0.26	−0.79** (0.09)	−1.06, −0.54		
Resilience → emotional expression → PTSS	−0.95 (0.13)	−1.21, −0.70			−1.92** (0.04)	−0.25, −0.08
Resilience → emotional processing → PTSS	−0.65 (0.15)	−0.95, −0.38			0.14** (0.04)	0.25, 0.08

CI, confidence interval; PTSS, posttraumatic stress symptoms. Unstandardized estimates are shown.

*p < 0.01, **p < 0.001.

## 4 Discussion

The present study found low levels of PTSS and depression and high levels of resilience among veterans, along with strong negative associations between resilience and symptoms. Moreover, these associations were mediated by emotional-approach coping. However, a distinct mediating role of each coping strategy emerged: Although both emotional expression and processing were associated with higher resilience, emotional expression mediated a positive association between resilience and PTSS, whereas emotional processing mediated a negative association with PTSS.

Like in previous studies (Fogle et al., 2020), veterans’ resilience levels were found to be relatively high and their PTSS and depression levels were generally low. Because Israel constantly deals with security threats, health care experts have highlighted the importance of the ecological construct of Israeli resilience. Composed of four major systems (micro or individual, meso or family, exo or society, and macro or state), resilience in Israel exists during times of routine life and emergency—and equally importantly, in the periods between the two (Nuttman-Shwartz and Green, 2021). The relations among these resilience levels are apparently relatively strong and stable in Israel, as described by experts who have highlighted Israeli’s “sense of mission” and “connectedness to others” as main resources that maintain their “inner strength, coping skills, and hope” (Corzine et al., 2017, p. 5). The findings of the current research are in line with this approach. Also, the strong association between PTSS and depression accords with previous studies (Armenta et al., 2019), suggesting that PTSD does not cover all manifestations of postservice distress and despite its centrality in the trauma discourse, represents only part of veterans’ posttraumatic emotional burden. The relations between PTSD and posttraumatic depression are complex and deserve further longitudinal research.

The current study was the first to examine the role of emotional-approach coping in relation to resilience and veterans’ PTSS. Structural equation modeling revealed that although both emotional-approach coping strategies were associated with higher resilience, they showed an inverse mediation effect. Emotional expression was negatively associated with PTSS, indicating that higher resilience was related to lower PTSS when connected with higher emotional expression (Van Voorhees et al., 2018; Fogle et al., 2020). This finding suggests that more use of emotional expression might enable emotions to be seen and shared with others, hence promoting relief (Stanton et al., 2000a). In line with Stanton et al.’s (2000a) findings, the analysis revealed that emotional processing was positively related to resilience but also higher PTSS. This finding suggests that higher involvement in emotional processing might increase PTSS among veterans, because it may represent a ruminative component.

Previous studies presented relations between the use of emotional-approach coping and lower levels of psychological symptoms regarding several stressful conditions, mostly concerning physical illness and physical pains (Austenfeld and Stanton, 2004; Jensen-Johansen et al., 2013; Batenburg and Das, 2014; Yeung and Chow, 2019). However, the clinical presentation of this trend seems to be more complex, with some research highlighting the fact that emotional processing’s effect may vary depending on the time since the traumatic or stressful event and how long this coping method is used (Stanton and Low, 2012; Juth et al., 2015). Similar to the present study’s findings, Hoyt et al. (2020) found that among older people, only emotional expression was related to better and more stable health outcomes over time, whereas emotional processing was related to increased depression in times of stress. Also, in support of the present study, only processing was found to be related to increased negative cognition and emotions (Watkins, 2004). These findings raise the questions of how, when, and for whom emotional-approach coping may be beneficial and whether it can be harmful in certain circumstances. A possible explanation for the sometimes-negative impact of intensive use of emotional processing is that it may foster rumination through the tendency to focus on negative emotions in a repeated manner, amplifying the consequences of those negative emotions. Rumination was found to be associated with increased symptoms of depression and anxiety and the onset of major depressive episodes (Nolen-Hoeksema and Davis, 1999; Vine et al., 2019). However, due to the correlational nature of the results, it may be that individuals with higher PTSS are more involved in emotional processing that encourages rumination.

Furthermore, veterans who presented high levels of emotional processing alongside high levels of resilience might experience resilience as a double-edged sword (Adler and Sowden, 2018). In other words, it seems that although these resilient veterans managed to process their emotions in a more thorough way alone, they were also vulnerable. Paradoxically, the ability to process distress alone can impair the possibility of reducing that distress. This finding is an example of veterans’ radical implementation of the army’s values and culture, which encourage its members to embrace selflessness and personal courage (Adler and Sowden, 2018), which might be interpreted as contradictory to turning to others for help.

Our research contributes to the understanding of this complexity by shedding light on how these processes occur among veterans who deal with military-related traumatic stress, which is characterized by substantial avoidance of emotional sharing stemming from the nature of the trauma and the atmosphere of military service (Solomon, 2020). Because combat soldiers usually seek to live up to masculine codes of behavior and face negative stigma when these codes are not respected (O’Loughlin et al., 2020), sharing of emotions and emotional expressions of feelings seen as “soft” and “weak” is usually difficult. Understanding the nuances of this trend among veterans is of great importance, especially in light of the gap between different types of intimacy they represent. Although soldiers and veterans tend to maintain very intimate relationships with their fellow squad members, which is expressed in the ability to share significant and powerful emotional experiences related to combat and service, they often simultaneously have difficulty sharing personal feelings or moments of emotional weakness and hardship arising from these same experiences.

The fact that emotional expression mediated PTSS for veterans with high resilience levels in this study might indicate the need to encourage veterans to share their hardships via social interaction. It also supports the important role of family members and close others in such sharing initiatives, because they may foster this positive trend and help break the loneliness cycle that sometimes traps trauma victims (Shorer et al., 2024).

### 4.1 Limitations

Our study has several methodological limitations. The research sample consisted of participants who applied to a program aiming to process combat experiences. It is reasonable to believe that they were more oriented toward emotional processing or expression of feelings than veterans who did not participate in such an intervention. Hence, our sample might be somewhat imbalanced. However, the large sample included full squads and companies from various army units. This means that people of varied backgrounds and many veterans who were not “emotionally oriented” participated in this intervention and study, and their standpoints might have balanced the sample.

Due to the cross-sectional design, the assessment of both resilience and PTSS levels should be interpreted cautiously, because the levels of these factors may change during post-trauma recovery and developmental stages (Masten, 2015). Moreover, as multilayered phenomena, both conditions should be further studied through the use of varied assessment tools and longitudinal research designs. Based on a limited cross-sectional design and only one resilience assessment tool, our study could not offer any causal interpretations or further explore distinct associations between specific resilience features and PTSS. Accordingly, this study does not permit causal inferences, and further studies using longitudinal design are encouraged. We recommend that future work on emotional-approach coping explore the relations between different emotional involvement and coping mechanisms among various trauma-exposed populations, along with studying the point at which emotional processing coping may become negative.

Despite the high rates of female soldiers in the Israeli military (Israel Defense Forces, 2013), few of them turn to military-experience processing programs, and units that use this psychological source are usually male dominated. One possible explanation for this is that female soldiers are expected to adopt “manly” trends during their service; hence, they learn to hide their emotions. Because emotional processing is the opposite of this, they might be confused by this ambiguous message (Harel-Shalev and Daphna-Tekoah, 2020). Because the current research dealt with male veterans only, its conclusions should not be generalized to female veteran populations.

### 4.2 Clinical applications

The results of the current study suggest that although resilience is a major factor in facing the effects of psychological trauma, its implementation and manifestations depend on individual coping skills. Common coping strategies should be examined in relation to the context in which they are implemented. Promoting resilience among veterans should encourage emotional expressions, and not limited to cognitive or emotional processing only. Interventions to promote the use of this strategy may include initiating social relationships, teaching stress management, and the restructuring of cognitive standpoints toward emotions (Van Voorhees et al., 2018). According to our findings, supporting emotional expression might reduce postservice emotional distress while not harming veterans’ resilience levels.

## 5 Conclusion

This study explored associations among veterans’ resilience levels, emotional coping mechanisms, and PTSS levels. The findings highlight the importance of emotional expressions of trauma-related reactions to enhance veterans’ well-being. Further investigation of trauma survivors’ specific emotional coping mechanisms is needed.

## Data availability statement

The raw data supporting the conclusions of this article will be made available by the authors, upon contacting the corresponding author.

## Ethics statement

The studies involving humans were approved by Board of Ethics, Faculty of Social Welfare and Health Sciences, University of Haifa, Israel. The studies were conducted in accordance with the local legislation and institutional requirements. The participants provided their written informed consent to participate in this study.

## Author contributions

SS: Conceptualization, Investigation, Project administration, Writing – original draft, Writing – review & editing. MW: Conceptualization, Data curation, Investigation, Methodology, Writing – review & editing. LC: Data curation, Investigation, Writing – review & editing. DM: Conceptualization, Writing – review & editing. MC: Conceptualization, Data curation, Investigation, Methodology, Supervision, Writing – original draft, Writing – review & editing.
